# Replication Compartments of Eukaryotic and Bacterial DNA Viruses: Common Themes Between Different Domains of Host Cells

**DOI:** 10.1146/annurev-virology-012822-125828

**Published:** 2022-09-29

**Authors:** David M. Knipe, Amy Prichard, Surendra Sharma, Joe Pogliano

**Affiliations:** 1 Department of Microbiology, Blavatnik Institute, Harvard Medical School, Boston, Massachusetts, USA; 2 Division of Biological Sciences, University of California, San Diego, La Jolla, California, USA

**Keywords:** DNA virus, bacteriophage, infection, viral replication compartment, phage nucleus

## Abstract

Subcellular organization is essential for life. Cells organize their functions into organelles to concentrate their machinery and supplies for optimal efficiency. Likewise, viruses organize their replication machinery into compartments or factories within their host cells for optimal replicative efficiency. In this review, we discuss how DNA viruses that infect both eukaryotic cells and bacteria assemble replication compartments for synthesis of progeny viral DNA and transcription of the viral genome. Eukaryotic DNA viruses assemble replication compartments in the nucleus of the host cell while DNA bacteriophages assemble compartments called phage nuclei in the bacterial cytoplasm. Thus, DNA viruses infecting host cells from different domains of life share common replication strategies.

## 1. INTRODUCTION

Viruses sequester their replication machinery within the infected cell to concentrate the viral gene products for optimal efficiency of replication, to concentrate the limiting cellular factors needed for viral replication, and to exclude inhibitory cellular factors. These assemblies or structures function in multiple viral processes during infection including viral nucleic acid transcription and/or replication and assembly of progeny viruses. Eukaryotic RNA viruses replicate and assemble their genomes in association with membranes, in association with cytoskeletal elements, or within membrane invaginations. Cytoplasmic DNA viruses, such as the poxviruses as described in the Greseth & Traktman review in this volume (1), replicate their genomes in viral factories or viroplasms. Nuclear DNA viruses replicate their genomes within defined areas of the eukaryotic cell nucleus called replication compartments (RCs), replication centers, or factories. DNA bacteriophages, which replicate in bacteria, have also been observed to compartmentalize their genomes, with certain phages forming structures called phage nuclei. In this review, due to length constraints, we focus on a comparison of the RCs of nuclear DNA viruses and DNA bacteriophages. We apologize for not being able to cite all the available literature on DNA virus RCs due to length constraints. We refer the reader to several articles that have reviewed DNA virus RCs more generally (2–4).

As prototypic examples of the RCs formed by bacteriophages and eukaryotic viruses, we focus on the DNA virus herpes simplex virus (HSV) and the jumbo bacteriophage φKZ family. Both have double-stranded DNA (dsDNA) genomes and a capsid protein with a specific protein fold called the HK97 fold (5), so they are classified into the same two viral taxonomic groups at the highest level, the *Duplodnavira* realm and the *Heunggongvirae* kingdom. However, the two viruses are very divergent with HSV in the *Herpesvirales* order (herpesviruses), while the Phikzviruses are in the *Caudovirales* order (tailed bacteriophages). Herpesviruses and tailed DNA bacteriophages also share mechanisms of virion assembly, maturation, and genome packaging (6), providing further evidence of a common evolutionary ancestor. In this review, we discuss how they also share the property of assembling subcellular compartments for replication and transcription of their DNA virus genomes, extending this property across viruses infecting different domains of host cells.

## 2. REPLICATION COMPARTMENTS IN EUKARYOTIC NUCLEAR-REPLICATING DNA VIRUSES

### 2.1. Herpes Simplex Virus

HSV undergoes a lytic, productive infection of epithelial cells and fibroblasts in which immediate-early (IE) proteins are expressed first and activate the expression of early (E) viral gene transcription. E proteins are largely involved in replicating the viral DNA, and late (L) genes are then transcribed to yield L proteins that assemble progeny virions. The virus spreads to sensory neurons where it establishes a latent infection, which can later reactivate to shed virus.

#### 2.1.1. Herpes simplex virus replication compartments as an example of eukaryotic DNA virus replication compartments.

**Figure 1.**
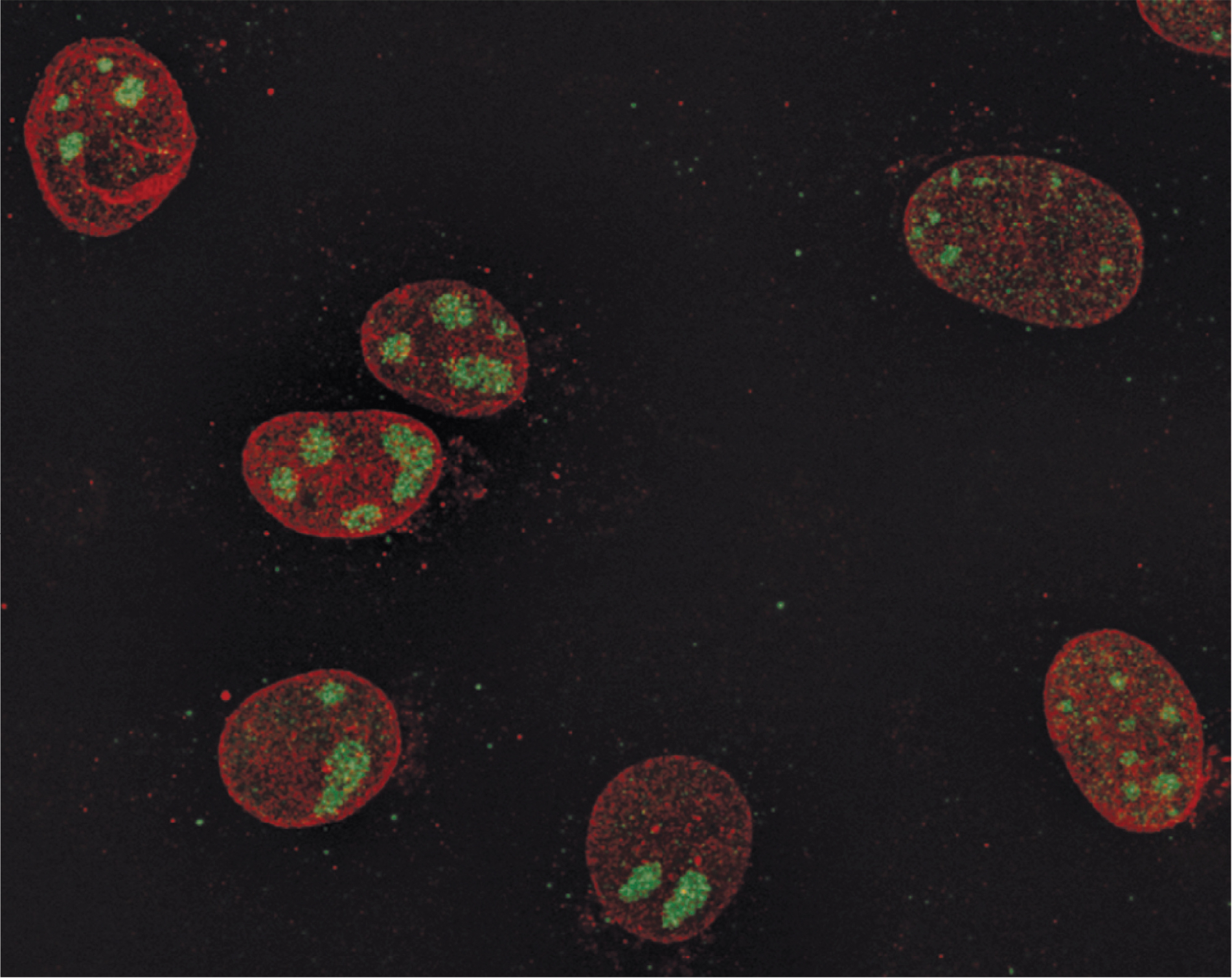
HSV RCs in the nuclei of infected cells. Shown are Vero cells infected with HSV-1 virus and fixed at 7 hpi and stained with anti-ICP8 antibody (*green*) to mark RCs and antilamin B1 antibody (*red*) to mark the nuclear lamina and define the boundaries of the nucleus. The punctate structures containing ICP8 within the larger globular RCs colocalize with sites of viral DNA synthesis. Abbreviations: hpi, hours post infection; HSV, herpes simplex virus; ICP, infected cell protein; RC, replication compartment. Copyright Lynne Chang and David Knipe.

HSV nuclear inclusion bodies and RCs have been studied extensively and therefore serve as the background for this discussion. Nuclear inclusion bodies in virus-infected tissues and cells were observed many years ago as structures that showed specific staining properties within infected cells and tissue, and these were used for diagnostic purposes. For example, cells and tissues infected with HSV showed intranuclear inclusions (7) or ground-glass structure-appearing structures in the nucleus that stain with basophilic or eosinophilic stains and sometimes are surrounded by a clear halo separating them from marginated chromatin (8). Electron microscopic studies of HSV-infected cells showed intranuclear inclusions made of a finely granular electron-dense material surrounded by a clear zone of nucleoplasm with clumps of material that stain with osmium stain around this near the nuclear envelope (9). Thus, there was consistent evidence from light and electron microscopy of nuclear inclusions in HSV-1-infected cells in culture and in human infected tissues. There was disagreement, however, about whether the nuclear inclusions were formed as part of the infection process or as the result of virus aggregation or cell degeneration (8). Localization of the HSV infected cell protein (ICP) 8 essential for DNA replication within the inclusions provided evidence for their important role in viral replication, and we termed them RCs (10) (Figure 1; see Section 2.1.3.2). RCs have now been well established to play a role in viral DNA synthesis, transcription, and DNA encapsidation.

#### 2.1.2. Formation of herpes simplex virus replication compartments.

**Figure 2.**
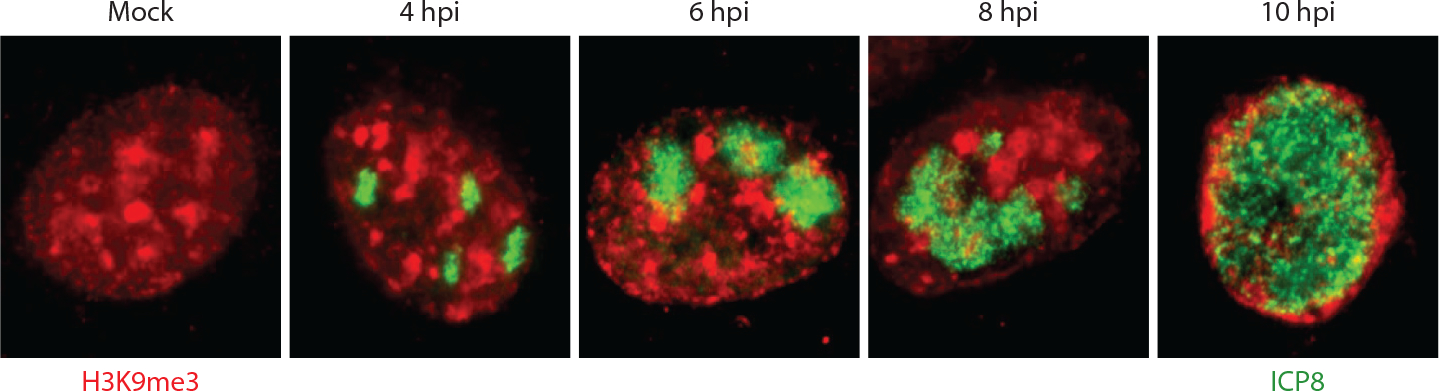
Heterochromatin is excluded from HSV replication compartments. Immunofluorescence images of human Hep-2 cells infected with HSV-1, fixed at the indicated hpi, and stained with anti-H3K9me3-specific antibody (*red*) and anti-ICP8 DNA-binding protein antibody (*green*). Abbreviations: hpi, hours post infection; HSV, herpes simplex virus; ICP, infected cell protein. Copyright Lindsey Silva and David Knipe.

The incoming viral DNA genome is transcribed to express first the IE gene products such as ICP4 and ICP0 that promote expression of the early gene products. The E proteins then assemble into replication complexes to replicate the viral DNA in RCs. Viral DNA replication is compartmentalized within the RCs, non-membrane-bound structures possibly assembled around the viral DNA molecules as discussed below, and the progeny viral DNA accumulates in these structures. As viral DNA synthesis proceeds, the RCs grow, move to nuclear speckles in the interior of the nucleus via an actin-dependent process (11), and merge to form large RCs (12, 13) (Figure 2).

To look at the molecular events in more detail, the incoming HSV DNA genome is targeted to a location near the nuclear periphery and/or nuclear lamina early after entry into the nucleus. The viral genome coming into the nucleus is recognized as foreign because it lacks histones, so cellular mechanisms rapidly load histones onto the viral DNA. These histones bear post-translational modifications that cause the compaction of the chromatin into a transcriptionally silent form termed heterochromatin (14, 15), as the host cell tries to epigenetically silence the foreign viral DNA, to prevent viral transcription. Host cellular restriction factors such as PML, ATRX, Sp100, and SUMO2, as well as IFI16, localize to input viral genomes within minutes after infection (16–18). These restriction factors result in increased heterochromatin on the viral genome (17, 19, 20), and ATRX (21) and probably IFI16 (22) maintain stable heterochromatin rather than load histones. In contrast, some have argued that the restriction factors are wrapped around the viral genome and sterically block transcription of the viral genome (16). Nevertheless, virion protein 16 (VP16) assembles a complex of host proteins and enzymes that removes the heterochromatin marks on histones at the promoters of IE genes and loads euchromatin marks (23, 24) to allow gene transcription. ICP0 is then expressed, and it acts as an E3 ubiquitin ligase to promote the proteasomal degradation of several host restriction factors that load and/or maintain heterochromatin on the HSV genome, resulting in a loss of heterochromatin on the viral DNA across the genome and allowing expression of ICP8 and the viral DNA replication proteins that form the RCs.

Prior to viral DNA synthesis or if viral DNA synthesis is blocked by inhibitors of the viral DNA polymerase, ICP8 and other viral proteins accumulate in numerous punctate sites through the nucleus that we call prereplicative sites (10). Some of these punctate structures are likely precursors of RCs. As viral DNA synthesis initiates, structures called early RCs, as detected by ICP8 immunofluorescence (IF) (25, 26), or genome complexes, as detected by ICP4 IF (12), then form near the inner nuclear membrane. Input HSV DNA was also reported to localize to nuclear domain 10 (ND-10) structures that contain PML (27, 28), and HSV RCs also form at prereplicative sites near ND-10 structures (29, 30). Later studies argued that ND-10 components such as PML protein localize to incoming genomes (31).

#### 2.1.3. Functions of herpes simplex virus replication compartments.

##### 2.1.3.1. Role for early replication compartments or genome complexes in immediate-early gene transcription.

The HSV-1 genome is found initially in genome complexes, which are defined as containing viral DNA and the IE protein ICP4, or small RCs near the nuclear periphery (12, 25, 26, 32). The localization of the genome to the nuclear periphery is linked to gene transcription because under conditions that lead to the release of RCs from the nuclear periphery [cells infected with a VP16 acidic activator domain mutant virus (32) or in mouse embryonic fibroblasts knocked out for lamin A/C (26)], viral heterochromatin is increased and viral gene expression is reduced. Thus, localization to the nuclear lamina or periphery could help ensure the transcriptional activation of viral genomes immediately after nuclear entry.

##### 2.1.3.2. Viral DNA synthesis.

Electron microscope autoradiography of HSV-infected cells labeled with ^3^H-thymidine showed intranuclear structures labeled with thymidine as potential sites for HSV DNA synthesis (33). IF detection of the HSV-1 ICP8 single-stranded DNA (ssDNA)-binding protein showed that it localizes to intranuclear globular structures (10) (Figure 1). Because ICP8 is required for HSV DNA replication (34), it was hypothesized that these were sites of viral DNA synthesis and the structures were called “replication compartments” (10). BrdU labeling of nascent DNA and in situ detection at times after infection when viral DNA synthesis was ongoing showed labeling of RCs (25), supporting the idea that viral DNA synthesis takes place in these compartments. Higher resolution microscopy showed that the punctate sites of ICP8 localization within the larger globular RCs colocalize with sites of BrdU labeling (35); therefore, ICP8 is located at sites of DNA synthesis within RCs.

HSV encodes seven proteins that are required for viral DNA synthesis, the HSV DNA polymerase holoenzyme consisting of the UL30 catalytic subunit and the UL42 processivity factor, the UL9 origin-binding protein, the ICP8 (UL29) ssDNA-binding protein, and the helicase-primase complex composed of the UL5, UL8, and UL52 proteins. These proteins are all localized to RCs (10, 25, 29, 36–39).

The role of host proteins in the RCs has also been investigated. Host proteins p53, pRb, replication protein A (RPA), RAD51, and NBS1 were first shown to localize to prereplicative sites and RCs by IF (40, 41). Proteomic studies (42) showed that ICP8 associates with and colocalizes in RCs with various cellular proteins including DNA replication or DNA damage repair proteins (RPA and MSH6), nonhomologous and homologous recombination [catalytic subunit of the DNA-dependent protein kinase (DNA-PK), Ku86, and Rad50], and histone modification and chromatin remodeling proteins (BRG1, BRM, hSNF2H, BAF155, mSin5, and histone deacetylase 2). The WRN helicase promotes viral replication while the Ku70 subunit of the DNA-PK reduces viral replication (42); thus, host factors in RCs both increased and decreased HSV-1 replication. The results in these previous papers were extended in a study (43) that showed that HSV infection activates a DNA damage response (DDR) in human cells that involves Mre11 and ATM. This study found that mutant human cells lacking ATM or Mre11 showed reduced HSV-1 replication relative to the same cells with the mutant proteins complemented. Different human cell types may have varying effects of DDR proteins because we found that ATM and Mre11 had little effect in knockout human fibroblasts on wild-type HSV-1 replication, but Mre11 restricted replication of an ICP0^−^ mutant virus (44). Direct roles of the DDR proteins in RC processes such as DNA synthesis remain to be defined.

HSV DNA synthesis involves the UL9 protein binding to one of the three viral origins of replication (oris), unwinding of the dsDNA by UL9 and the helicase primase complex (UL5, UL8, and UL52), binding of ICP8 to the ssDNA, and recruitment of the HSV DNA polymerase holoenzyme (UL30 and UL42) to replicate the viral DNA (45). Replication is postulated to occur by initiation on a circle to form a theta replicating structure followed by rolling circle replication or by initiation on a linear model followed by recombination, both processes yielding concatemeric progeny molecules. Transfection of the genes encoding UL5, UL8, UL52, and ICP8 is sufficient to form prereplicative sites (29, 30, 39), and transfection of six HSV DNA replication protein genes is sufficient to form RCs without an HSV ori sequence (30).

Despite extensive study, we do not yet know the molecular structure of HSV RCs, but they do increase in size in parallel with viral DNA synthesis (10, 25). EM analysis has shown that they are not membrane bound; thus, some have proposed that RCs are liquid-liquid phase-separated condensates promoted by ICP4 (46) based on the round structure of the initial RCs and the fluid-like properties of proteins within the RCs. In fact, ICP4 is required for the expression of the viral proteins involved in DNA synthesis and RC formation, so it is hard to parse out a separate function for ICP4 in the structural properties of RCs. Furthermore, the RC might not be a condensate but rather a structure formed by proteins binding to the accumulating progeny viral genomes (47), an idea supported by our observation of columnar structures in RCs that could be built on viral DNA (48). Clearly, this is an important area for future study.

Using pseudorabies virus recombinants, a close relative of HSV, that express various fluorophores, Kobiler et al. (49) showed that fewer than seven incoming viral genomes initiate infection within an individual cell. This approximated the number of RCs in HSV-infected cells (13, 25, 50); thus, there may be limited numbers of genome attachment sites for initiation of infection. A study with similar HSV recombinants showed that an HSV RC can initiate with a single viral genome and concluded that coalescence between RCs allowed recombination between coinfecting genomes (51). It remains to be proven that the areas of overlap are truly recombinant genomes versus mixing of the two parental genomes. If recombination between coinfecting genomes occurs only when RCs merge, this would be after significant DNA synthesis had occurred. There may be ways for exchange of viral genomes between RCs other than their physical merger to explain the observed high efficiency of recombination between coinfecting HSV strains.

RCs are thought to compartmentalize and concentrate the components for viral DNA synthesis, but no direct experimental evidence has been obtained to document this. RCs appear to exclude histones (52) (Figure 2) in that as the RCs grow, they push host chromatin to the periphery of the nucleus and cause the classical margination of host chromatin. The mechanism of this effect remains to be defined, but it is tempting to speculate that the viral genomes and associated protein may displace host chromatin from its normal intranuclear binding sites. RCs also appear to exclude heterochromatin (26) (Figure 2), but this is largely due to VP16 and ICP0 promoting removal of heterochromatin from the viral genome. In the absence of ICP0, IFI16 assembles filaments of restriction factors in RCs (53). These filaments appear to exert a repressive effect on all RCs within the nucleus of the cell, and this *trans* effect may represent a novel mechanism within and between the RCs.

##### 2.1.3.3. Late gene transcription and RNA export.

The HSV ICP4 transcriptional activator protein (54, 55) and cellular RNA polymerase II (Pol II) (56) localize to RCs, consistent with the idea that transcription from progeny viral DNA molecules occurs in RCs. RNA pulse labeling also shows that viral transcription takes place in RCs at late times (50). The HSV ICP27 protein, which stimulates L gene transcription, localizes to RCs as shown by RNA pulse labeling (57). ICP27 interacts with the C-terminal domain of cellular RNA Pol II (58, 59) and with the HSV ICP8 SSB protein (60). Based on these interactions, ICP27 was hypothesized to recruit RNA Pol II to L gene promoters (60). ICP27 mutants that are specifically defective for L gene transcription localize to RCs but do not recruit RNA Pol II (59), supporting the idea that ICP27 recruits RNA Pol II to RCs.

HSV infection inhibits RNA splicing (45), which is part of the normal cellular pathway for RNA export, so the mechanism of nuclear export of HSV RNAs, nearly all of which are unspliced, from the cell nucleus remains to be explained. Chang et al. (11) observed that small RCs move by directed motion promoted by actin and myosin and enhance the export of late viral messenger RNAs (mRNAs) (11).

##### 2.1.3.4. DNA encapsidation.

The major capsid protein, ICP5, and filled and empty capsids are localized to RCs (35). Capsid assembly and genome encapsidation take place at early and late times of infection within RCs at sites near those of viral DNA replication and at later times, perhaps also in nuclear structures called assemblons in certain cell types. Thus, encapsidation is likely coupled to viral DNA synthesis in RCs.

### 2.2. Other Herpesviruses

RCs with many of the same properties are formed by other human herpesviruses, including Epstein-Barr virus (61), human cytomegalovirus (62), varicella-zoster virus (63), human herpesvirus 6 (64), and Kaposi’s sarcoma herpesvirus (65).

## 3. OTHER NUCLEAR-REPLICATING DNA VIRUSES

### 3.1. Human Adenoviruses

Human adenovirus (HAdV) RCs appear as electron-dense viral inclusion bodies in the host cell nucleus using transmission electron microscopy, and they serve as sites for viral genome replication, viral gene transcription, and viral mRNA splicing. Adenoviral DNA has been detected in RCs by fluorescent in situ hybridization (FISH) and by detection of DNA synthesis with ^3^H-thymidine or nucleoside analogs (66). Early electron microscopic studies showed that compact fibrillar early replicative sites called ssDNA accumulation sites (ssDASs) appear with the onset of viral DNA replication and contain the viral ssDNA DNA-binding protein (DBP) (67, 68). As viral DNA replication progresses, fibrillo-granular replicative sites called the peripheral replicative zone (PRZ) are arranged in ring-like structures at the periphery of the ssDAS. The PRZ serves as the major site for viral DNA synthesis and was also suggested to be the site for viral transcription using RNA-FISH and nascent RNA radiolabeled with ^3^H-uridine (69). However, pulse-chase experiments argued for spatial separation of HAdV replication and transcription, where newly synthesized viral DNA moves from the PRZ to the adjacent and distinct sites in nucleoplasm that support RNA transcription and processing (70). IF staining of the DBP revealed that early RCs contain numerous small DBP foci, which coalesce to form larger DBP foci or late RCs (67).

The presence of the viral genome packaging protein L1 and histone-like viral core protein VII in the PRZ in RCs suggested that RCs may serve as sites for initial assembly of the virus core (71). In late RCs, replicated DNA accumulates in a subcompartment termed the virus-induced post replication (ViPR) body that was proposed to be involved in genome replication and packaging (72). However, the absence of capsid proteins pVI and IX in ViPR bodies suggested that ViPR bodies may not be sites of encapsidation (73).

### 3.2. Parvoviruses

Parvoviruses are dependent on either coinfection with helper viruses or host cell functions for their DNA synthesis. Thus, adeno-associated viruses (AAVs) hijack RCs of a helper virus, adenovirus (AdV) or HSV, to utilize their viral proteins for their own DNA replication (74–76). The AAV Rep78 and Rep68 proteins are essential for viral DNA synthesis and are the most frequently used markers for AAV RCs. The autonomous H-1 parvovirus is not dependent on coinfection with another DNA virus and assembles RC-like structures called autonomous parvovirus-associated replication bodies, which are electron-dense foci containing the viral NS1 initiator/helicase/transcriptional activator protein essential for viral DNA replication (77). Incorporation of BrdU at these RCs supports the idea that viral DNA replication occurs in these sites. Parvovirus minute virus of mice hijacks sites of cellular DNA damage preloaded with DNA repair/replication proteins to establish RCs and replicate and transcribe its genome (78, 79).

The nature and composition of RCs are determined by the type of parvovirus and virus coinfecting the host cell. For example, while AAV RCs associate with PML-NBs in AdV-infected cells, this was not observed in HSV-1 infected cells in which PML is targeted for degradation by HSV-1 ICP0 (74). Autonomous parvovirus RCs are devoid of PML-NBs (77). Despite the presence of a DNA-dependent protein kinase catalytic subunit (DNA-PKcs) at RCs formed by the coinfection of AdV and AAV and the finding that DNA-PK is the major activated DDR signaling pathway, there is no defined role for DNA-PK signaling in driving AAV replication (80).

### 3.3. Papillomaviruses

Papillomaviruses (PVs), which are dsDNA viruses that cause warts, establish a latent infection in basal epithelial cells, and the genome persists as an unintegrated episomal molecule in these cells. Upon differentiation of these cells into keratinocytes, PVs undergo a productive infection and produce infectious virus. Differentiation-dependent productive amplification of the PV genome occurs in defined nuclear compartments or RCs. RCs are foci of active DNA replication marked by two essential early PV viral proteins with dedicated functions in human PV (HPV) genome replication, the E1 DNA helicase/origin-binding protein and the E2 transactivator/transrepressor/episome segregator (81–83). Localization of the L1 major capsid protein and the L2 minor capsid protein to RCs suggested that the RCs are sites of assembly of progeny PVs (84–86). Compared to other nuclear DNA viruses, the molecular mechanism of productive HPV amplification is not well elucidated, largely due to HPV’s need for differentiated keratinocytes such as organotypic epithelial raft cultures for productive infection (87, 88).

Host proteins that are frequently found in RCs induced by other DNA viruses are also found in PV RCs, including PML, DAXX, and Sp100. While PML has an antiviral role with other DNA viruses, it was found to enhance PV nuclear retention and transcription (89, 90). Sp100 colocalizes with E1 and E2 in PV-induced RCs, where it acts as a viral restriction factor for HPV by blocking productive HPV replication in differentiated cells (82). Expression of L2 protein was reported to reorganize ND-10 bodies by recruiting DAXX and dispersing Sp100 while having no effect on PML (91). A key study illustrated that the activation of cellular DDR proteins in ATM and ATR pathways was required for both differentiation-dependent HPV genome amplification and efficient formation of HPV RCs (92). Localization of cellular homologous recombination/repair proteins, phosphorylated RPA, Rad51, and BRCA1 was also found in HPV RCs (93).

### 3.4. Polyomaviruses

Genome replication of simian virus 40 (SV40), a polyomavirus (PyV), occurs in defined RCs near ND-10 structures (27). While DNA replication of SV40 is restricted to RCs near ND-10, SV40 transcription is observed throughout the nucleus, including RCs (94). Live cell microscopy with green fluorescent protein (GFP)-tagged host RPA revealed that RPA colocalizes with viral DNA and the viral large T antigen (LT) from murine PyVs (MuPyVs) at RCs, and RPA foci increase in size over the course of infection (95). Furthermore, it was found that small T antigen (ST) has a role in RC expansion but not in RC formation. Higher resolution study of MuPyV RCs by 3D structured illumination microscopy revealed the existence of two subdomains: a replication-associated subdomain containing LT, nascent viral DNA, and dim RPA32 signal, and a repair-associated subdomain containing focal RPA32 signal, phospho-ATM, and viral DNA (96).

PML-NBs and/or their components can be either restrictive as with JC virus (JCV) replication or nonrestrictive as with BK virus (BKV) replication (97, 98). BKV counteracts PML-NBs through reorganization of PML-NBs by dispersing two resident components, Sp100 and DAXX (98). However, JCV is incapable of modulating PML-NBs, and PML-NBs exert a restrictive effect (97). An ultrastructural study showed that PML is dispensable for the formation of RCs and viral replication of MuPyVs in both in vitro and in vivo studies (99). This study also reported that the absence of PML had no effect on the colocalization of Mre11, LT, and PyV DNA. PyVs manipulate both the ATM and ATR arms of DNA damage signaling (100, 101). Activation of the ATM kinase signaling pathway plays a vital role in supporting SV40 replication through the recruitment of T antigen and gamma-H2AX proteins at RCs and degradation of Rad50 or Nbs1 in the Mre11-Rad50-Nbs1 complex (102).

## 4. REPLICATION COMPARTMENTS IN BACTERIAL DNA VIRUSES

### 4.1. Nucleus-Forming Phage

Only recently have we discovered that some bacteriophages also form RCs. The most remarkable example of this is the formation of a nucleus-like structure recently described for a handful of jumbo phages, which are loosely defined as any phage with a genome larger than 200 kbp. Below we summarize the various steps of the nucleus-forming phage replication pathway, focusing on the best-studied *Pseudomonas* jumbo phage. Briefly, over the course of the lytic cycle, phage-encoded proteins completely restructure the bacterial cell to establish an intricately organized viral replication factory (103–107). Multisubunit RNA polymerases provide temporal regulation of gene expression, allowing complex structures to form in the appropriate time window during the lytic cycle (111–114). Immediately after injection of DNA into the host cell, a proteinaceous shell is formed that encloses the phage DNA to form a structure known as the phage nucleus (103, 105, 106). One of the first structural changes to occur to the host cell is the degradation of the bacterial chromosome (103–107), permanently eliminating the host’s transcriptional response and providing space for the creation of a unique viral RC. A tubulin-based cytoskeletal filament (PhuZ) organizes the replication machinery by centering and/or rotating the phage nucleus to allow efficient packaging of capsids (103–105, 107). Late during infection, mature virions form spherical assemblages, named bouquets, at specific locations in the cell (125). Below we describe the nucleus-forming replication pathway and compare it with the replication factories formed by other phage.

#### 4.1.1. Phikzviruses: *Pseudomonas* jumbo phages 201φ2-1, φKZ, and φPA3.

The phage nucleus was originally discovered through studies of *Pseudomonas chlororaphis* phage 201φ2-1 (103, 104, 108). Later, *Pseudomonas aeruginosa* phages φKZ (111) and φPA3 (109) were shown to replicate using a similar life cycle (105, 106). Upon phage particle binding to the host cell, phage DNA is injected into the cytoplasm near the cell pole (103–107). Proteins important for the initial events of establishing viral replication are contained within the phage particle and are thought to be injected along with the phage DNA (108, 110, 111). Some of these injected proteins make up a virion-associated multisubunit RNA polymerase complex (virion RNAP) that is thought to transcribe genes that are expressed early in the lytic cycle (108, 110, 111, 114). A second multisubunit RNA polymerase that is not associated with the mature viral particle (nonvirion RNAP) (112, 113) is expressed as part of the early program of gene expression (114). Together, these two polymerases are responsible for temporal regulation of genes during the infection cycle (114).

#### 4.1.2. Formation of the phage nucleus.

**Figure 3.**
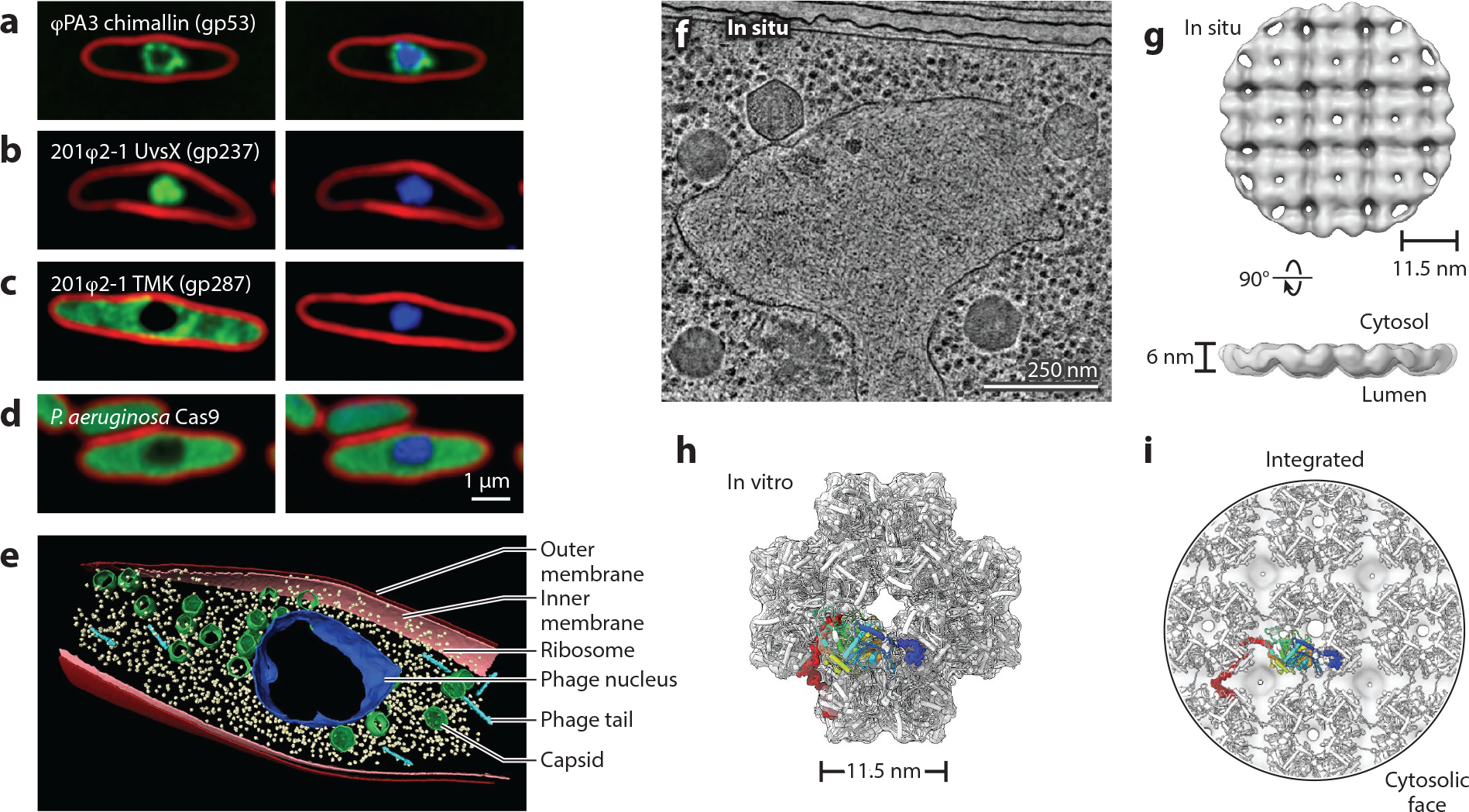
Images of *Pseudomonas* infected with nucleus-forming jumbo phage, illustrating the subcellular localization imposed by formation of a nucleus-like replication compartment. Membranes are stained with FM4-64 (*red*), and DNA is stained with DAPI (*blue*). (*a*) Shell protein tagged with GFP (*green*) surrounds replicating phage φPA3 DNA in *Pseudomonas aeruginosa*. (*b,c*) Phage 201φ2-1 proteins tagged with GFP in *Pseudomonas chlororaphis* involved in DNA processes such as gp237 (recombination-related protein UvsX) colocalize with phage DNA (*b*) while metabolic enzymes such as gp287 (TMK) (*c*) are excluded. (*d*) An example of a host Cas9 enzyme tagged with GFP in the cytoplasm of *P. aeruginosa* being excluded by the φPA3 phage nucleus. The scale bar is 1 micron. (*e*) Segmented cryo-electron tomogram of focused ion beam-milled phage 201φ2-1 infected *P. chlororaphis* showing chimallin protein (*blue*), ribosomes (*yellow*), phage tails (*cyan*), inner membrane (*pink*), outer membrane (*red*), and capsids (*green*). (*f*) Cryo-electron tomogram of *P. chlororaphis* cell infected by 201φ2-1 jumbo phage. The scale bar is 250 nm. (*g*) In situ subtomogram average (EMD-25221) of the native 201φ2-1 ChmA lattice derived from cellular tomograms like the one presented in panel *f*. (*h*) In vitro single-particle cryo-electron microscopy structure of purified, recombinant ChmA assembled as a cubic 24-mer (PDB-7SQQ, EMD-25390). The coordinate model is depicted as an illustration and the map as a transparent surface. The structure is depicted along the fourfold axis. An individual ChmA protomer is rainbow colored from the N to C termini, and the extended N-terminal and C-terminal segments are depicted as spheres. Unresolved loops connecting the terminal segments are modeled for visualization purposes. (*i*) ChmA lattice model derived from combining the in situ map (transparent surface) and in vitro coordinate model (illustration) shown from the cytosolic face. An individual protomer is colored sequentially as in panel *h* with the extended terminal segments depicted as spheres. Abbreviations: GFP, green fluorescent protein; TMK, thymidylate kinase. Panels *a* and *d* copyright 2022 Amy Prichard and Joe Pogliano; panels *b* and *c* copyright 2022 Vorrapon Chaikeeratisak and Joe Pogliano; panels *e* and *f* copyright 2022 Thomas Laughlin and Elizabeth Villa; panels *g*, *h*, and *i* copyright 2022 Thomas Laughlin, Kevin Corbett, and Elizabeth Villa.

The phage nucleus was discovered by visualizing the subcellular localization of phage proteins within infected cells using GFP fusions (103) (Figure 3a–d). The most abundant protein made early upon infection appeared to surround and fully enclose replicating phage DNA in a proteinaceous compartment, which was termed the phage nucleus (103, 105) (Figure 3a). Further studies using GFP fusions showed that phage proteins involved in DNA processes, including DNA and RNA polymerases, DNA ligases, and recombination-related proteins, colocalize with phage DNA inside the phage nucleus (Figure 3b), while ribosomes and proteins that perform metabolic functions, such as thymidylate kinase, localize in the cytoplasm (103, 105) (Figure 3c). Phage mRNA is synthesized inside the phage nucleus and subsequently transported to the cytoplasm, where it is translated into protein by ribosomes. Phage proteins involved in DNA processes are selectively imported into the phage nucleus while other proteins are excluded. This two-way exchange of mRNA and proteins suggests that the phage nucleus contains pores through which these molecules pass (103, 105). Although the phage nucleus is structurally distinct from the eukaryotic nucleus, its function is analogous: The phage nucleus separates DNA from metabolic enzymes and ribosomes in the cytoplasm, exports mRNA, and selectively imports proteins.

The molecular architecture of the phage nuclear shell was discovered by combining cryo-electron tomography of infected cells with high-resolution cryo-electron microscopy of purified proteins (115). These studies showed that the phage nucleus is composed of a single ~6-nm-thick layer of the major nuclear shell protein (Figure 3a, e–g). This protein was termed chimallin (ChmA) after *chimalli*, a shield carried by ancient Aztec warriors, due to its ability to shield phage genomes from host defenses (116–118). ChmA forms a flexible, single-layer-thick lattice through interactions of its extended N and C termini with neighboring protomers (115, 124) (Figure 3f–i). The pores in the ChmA lattice measure only ~2 nm in width, too small for the passage of most proteins and likely too small to pass viral mRNAs. This observation implies that additional minor shell components likely incorporate into the ChmA lattice to mediate mRNA export, specific protein import, and phage capsid docking and filling with genomic DNA (115). The identification and characterization of these minor shell components will be required for a full understanding of the architecture and function of the phage nucleus.

#### 4.1.3. *Serratia* phage PCH45 and *Pseudomonas* phage φKZ evade bacterial defenses.

**Figure 4.**
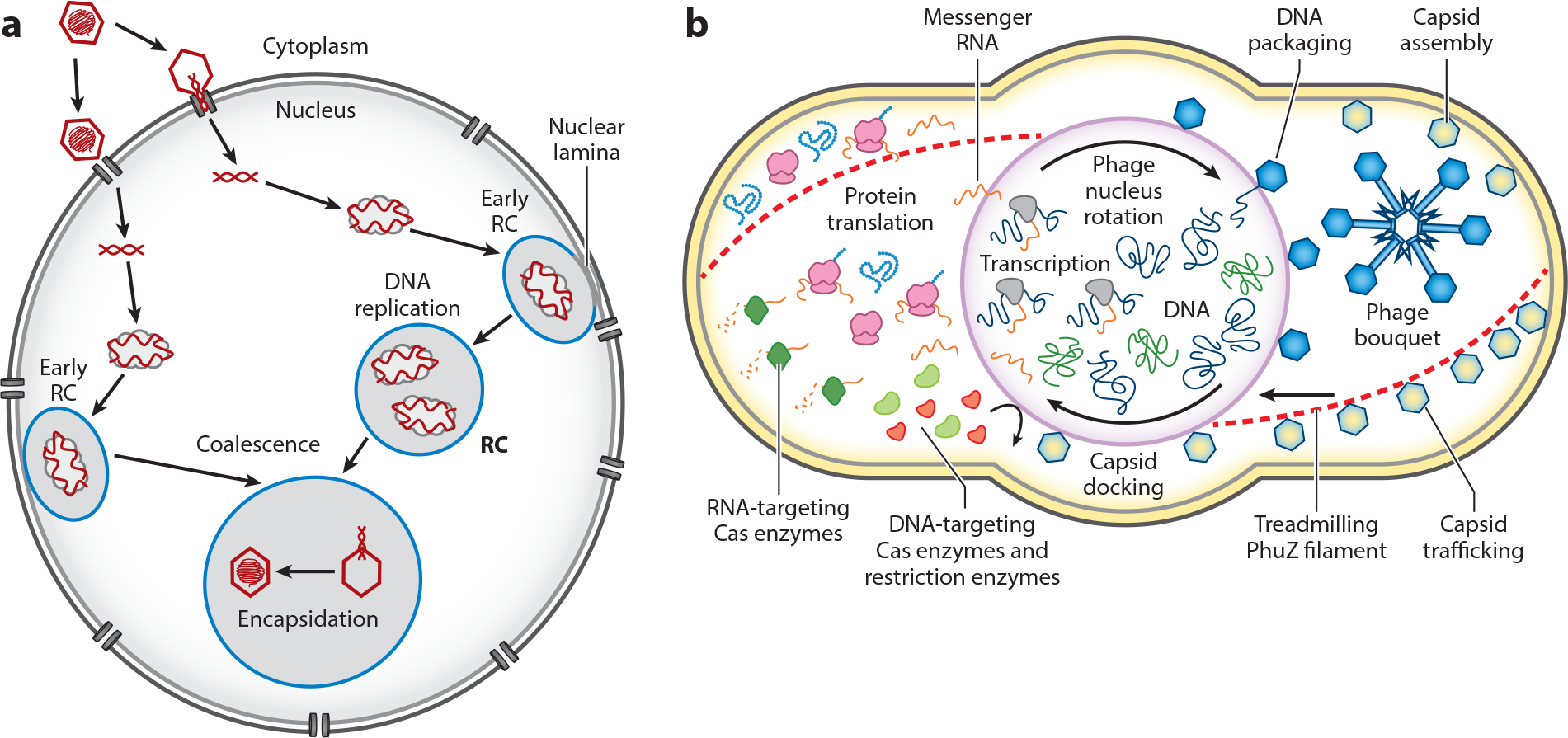
Comparison of RCs formed in herpes simplex virus–infected cells (*a*) and a phage nucleus and associated structures formed in nucleus-forming jumbo phage–infected *Pseudomonas* (*b*). Abbreviation: RC, replication compartment. Panel *a* copyright Surendra Sharma and David Knipe; panel *b* copyright 2022 Amy Prichard and Joe Pogliano.

One of the benefits of replicating in a compartment may be as a strategy to minimize attack from host defense mechanisms. In nucleus-forming phages, restriction enzymes and Cas enzymes are excluded from entering the phage nucleus (116–118) (Figures 3d and 4b). The phage nuclei of both *Serratia* phage PCH45 and *Pseudomonas* phage φKZ protect phage DNA from attack by DNA-targeting Cas enzymes and restriction enzymes. However, these phages are still susceptible to RNA-targeting Cas13 because it acts on the cytoplasmic RNA (116, 117) (Figure 4b).

#### 4.1.4. Role of the spindle apparatus in centering the phage nucleus.

Another key protein involved in nucleus-based phage replication is a phage-encoded tubulin homolog known as PhuZ (pronounced *fuzz*) (104, 105, 107, 119, 119a). PhuZ monomers of 201φ2-1 polymerize to form polarized, triple-stranded filaments with plus and minus ends that have distinct dynamic properties (104, 107, 119a). PhuZ filaments play several key roles in organizing phage replication within the host cell including positioning and rotating the phage nucleus and delivering capsids to it (103, 105, 120). In *Pseudomonas* phages 201φ2-1, φKZ, and φPA3, PhuZ filaments form a bipolar spindle that extends from each pole of the cell toward the phage nucleus (103–105, 107). Early during infection, the plus ends of the filaments undergo cycles of growth and shrinkage, known as dynamic instability, by adding or removing monomers (107). The minus ends appear to be located at the poles, with the plus ends oriented toward midcell where they provide periodic pushing forces to position the phage nucleus near the cell midpoint (107). Dynamically unstable filaments can move objects through the bacterial cell, and if they have the proper orientation and kinetic properties relative to cell size, they can position objects at midcell (121). This property of dynamic instability is shared with eukaryotic microtubules, which position chromosomes in the center of the cell during mitosis and meiosis.

#### 4.1.5. Capsid trafficking and phage nucleus rotation.

After capsids are assembled, they attach to the PhuZ spindle and are trafficked toward the phage nucleus via treadmilling of the filaments (120) (Figure 4b). Treadmilling occurs when subunits add to one end of the filament near the cell pole while simultaneously depolymerizing from the end adjacent to the phage nucleus (120). After being transported, capsids dock on the surface of the phage nucleus and package phage DNA, which allows the DNA to enter the capsids without being exposed to host defense nucleases in the cytoplasm. Capsids delivered to the phage nucleus are found spread across the surface of the compartment (103) due to PhuZ filament treadmilling, which provides pushing forces that cause the phage nucleus to rotate (120). Rotation allows for a more even distribution of the capsids across the surface of the phage nucleus (Figure 4b). While cargo trafficking along microtubules is a key feature of eukaryotic cells, it is currently the only known example of a tubulin-based filament involved in cargo trafficking in a prokaryotic cell.

Although the PhuZ cytoskeleton is unique to nucleus-forming jumbo phage, the reliance upon a cytoskeleton for movement and positioning is common for eukaryotic viruses. HSV-1 RCs can be positioned in the nucleus by actin filaments (11). Many eukaryotic viruses rely upon motor proteins to travel along host cell microtubules to reach preferred replication sites (122). In comparison, phage-encoded PhuZ filaments use treadmilling instead of motor proteins for cargo transport. Thus, while both eukaryotic and prokaryotic viruses use cytoskeletal filaments for intracellular movement, they do so by using distinct mechanisms.

#### 4.1.6. *Escherichia* phage Goslar forms a phage nucleus whose rotation is driven by a vortex of PhuZ filaments.

Like the previously discussed Phikzviruses, the recently discovered *Escherichia coli* jumbo phage Goslar also forms a phage nucleus that sequesters phage DNA and phage-encoded proteins within a proteinaceous shell away from ribosomes and metabolic enzymes in the cytoplasm (123). However, Goslar PhuZ filaments do not set up a bipolar spindle like Phikzviruses and do not position the phage nucleus at midcell but instead rotate the phage nucleus by forming a striking vortex-like structure that emanates out from its surface (123). This vortex provides radial pushing forces that drive phage nucleus rotation with a rotational velocity similar to that observed in Phikzviruses (120, 123). Thus, the distantly related nucleus-forming phage Goslar and the Phikzviruses have evolved two distinct ways to achieve the same function of phage nucleus rotation, which likely provides the conserved function of evenly distributing capsids on the phage nucleus surface, thereby facilitating DNA encapsidation. While PhuZ filaments and the process of rotation are conserved, dominant negative (104, 123) and gene knockout studies (123a) suggest that PhuZ is not essential for phage replication and instead increases the efficiency of progeny production by ~50% (104).

#### 4.1.7. Host cell wall remodeling.

Nucleus-forming jumbo phages typically expand the size of the cell to accommodate the phage nucleus. Goslar restructures the host cell wall by creating a large bulge centered around the phage nucleus, allowing it to achieve a diameter greater than the width of a typical *E. coli* cell (123). This results in infected cells having a unique morphology to accommodate the growing phage nucleus. The *Pseudomonas* cell is remodeled to form a central bulge that is 30% wider than the cell poles upon infection by Phikzviruses (103–105, 107). Formation of a large and sophisticated structure for viral replication in a cell as small as a bacterium thus involves both host cell shape remodeling and a cytoskeletal element for subcellular organization and efficient packaging of capsids.

#### 4.1.8. Phage bouquets.

After encapsidating DNA, capsids and tails assemble to form mature phage particles that are often localized in highly organized bundles known as bouquets because their tails face inward toward the center like the stems of flowers while their capsids face outward (125) (Figure 4b). Phage bouquets have been observed in *P. aeruginosa* phages φKZ and φPA3, and in *E. coli* phage Goslar, demonstrating that they are conserved (123, 125). φPA3 bouquets form at a specific distance from the phage nucleus, even in the presence of PhuZ mutations that misposition the nucleus, suggesting an active mechanism for their formation and positioning (125). While the purpose of phage bouquets is currently unknown, they might represent a different type of viral replication factory and serve as phage maturation compartments, where the tails assemble and attach to the capsids, or as storage sites for mature phages to keep the cytoplasm organized during replication.

### 4.2. *Escherichia* Phage *λ* Factories

Recently, *E. coli* phage *λ* has been shown to exhibit subcellular organization during its replication although its genome is not fully enclosed by a proteinaceous shell like the phage nucleus (126). After *λ* DNA is injected into a host cell, it begins replicating its genome in subcellular viral DNA replication complexes known as factories (126). These factories remain separate from each other due to the host bacterium’s chromosome preventing their interaction (126). This allows each viral factory to replicate individually and may influence recombination and competition between coinfecting phages because distant factories would be less likely to exchange genetic material and would be more likely to compete for resources, such as host enzymes required for DNA replication.

The individual nature of these *λ* factories means that the decision to form a lysogen or to proliferate via lytic replication is made independently by each infecting phage (126). Individual mRNA transcripts do not diffuse throughout the cell but remain localized with the phage DNA (126). Although coinfecting *λ* phages may have identical genomes, the physical separation of their replication factories can result in contradictory gene expression. This mosaic gene expression within a single bacterial cell culminates with a decision to become lytic or lysogenic based on the production of diffusible proteins (126). If enough cI repressor is produced early by one phage, it can turn off the expression of lytic genes of a second phage. Likewise, if the lytic program has proceeded far enough, it can lead to phage replication and cell lysis, killing the phage that had chosen to lysogenize.

### 4.3. Other Examples of Replication Compartments in Prokaryotes: *Bacillus* Phages SPP1 and φ29

Similar to *λ*, the lytic *Bacillus* phage SPP1 displays a subcellular localization pattern where phage DNA and replication machinery coalesce in viral factories that are not enclosed in a proteinaceous shell (127). However, it differs in that SPP1 frequently forms only one large RC that competes for space with the nucleoid. Mature viral particles accumulate in distinct regions from the RC and likely serve as storage and/or assembly sites for mature virions. The phenomenon of viral replication factories has also been reported for *Bacillus* phage φ29, which uses MreB to organize its DNA replication machinery at specific sites within its host cell (128, 129).

## 5. SUMMARY AND PERSPECTIVES

Viruses of both prokaryotic and eukaryotic cells share a conserved strategy of making factories in defined subcellular locations along with all of the proteins needed for their replication. Both eukaryotic DNA virus RCs and phage nuclei likely benefit viruses by concentrating the host and viral factors needed for viral replication and excluding restrictive host factors. Cytoskeletal structures are used by both eukaryotic viruses, in the form of host actin and the nuclear lamina, and some bacteriophages, in the form of phage-encoded PhuZ or host MreB, to localize RCs to specific domains of the infected cell. Phage that replicate by forming a nucleus stand out among these viruses for enclosing their RC in a proteinaceous shell, which may facilitate keeping DNA and proteins required for DNA replication and repair concentrated in the same space.

Additionally, it is worth considering how RCs contribute to viral speciation. The challenges and benefits of replicating inside an RC are different compared to cytoplasmic replication. Thus, selective pressures that might contribute to viral speciation have been studied in the context of nucleus-forming jumbo phages and eukaryotic viruses that replicate in RCs. First, the RC itself can serve as a barrier to genetic exchange. If two viruses infecting the same host cell make separate RCs, this would limit recombination between their genomes (130, 131). This phenomenon is known as subcellular genetic isolation and is a mechanism that allows viruses to diverge and retain their species identity even when they infect the same host cell (130). Second, divergent viral proteins may become virogenesis incompatibility factors if they interact nonproductively during coinfection (130). As an example, in the related *Pseudomonas* phages φKZ and φPA3, PhuZ homologs are similar enough to copolymerize but divergent enough to prevent the resulting filaments from treadmilling normally (130). This causes the spindle to be defective, which has previously been shown to result in a 50% decrease in progeny (104), highlighting that φKZ and φPA3 are likely different viral species. Virogenesis incompatibility mechanisms are not unique to nucleus-forming jumbo phages, however, and have been observed in segmented influenza viruses (132–134). Subcellular genetic isolation mechanisms have been studied in eukaryotic viruses such as herpesviruses (51) and poxviruses (131), which also form RCs during their replication cycles [see the Greseth & Traktman article in this volume (1)]. HSV-1 forms individual RCs inside the host cell’s nucleus, which may limit recombination between coinfecting viruses unless they coalesce (51). Vaccinia, a poxvirus, replicates in factories that are sometimes surrounded by membranes (131). In herpesviruses and poxviruses also, recombination would not be possible between viral genomes unless the RCs merge.

The commonality of these structures and mechanisms for many different types of DNA viruses across domains of life emphasizes the importance of compartmentalization. Further knowledge of their structure and function could lead to antiviral agents acting against a broad variety of eukaryotic viruses or development of more efficacious phage therapy treatments for bacterial infections.
